# The cow’s milk allergy that wasn’t: allergy to supplemental oral lactase enzyme

**DOI:** 10.1186/s13223-023-00809-z

**Published:** 2023-07-14

**Authors:** Sarah K. Lohrenz, A. Kanani

**Affiliations:** College of Medicine, University of British Columbia, Vancouver, BC Canada

**Keywords:** Anaphylaxis, Lactase-enzyme allergy, Supplement allergy

## Abstract

**Background:**

Allergy to supplemental lactase is sparsely reported in the literature with only one prior case of anaphylaxis documented [2]. Reactions to this agent can occur following cow’s milk ingestion which could lead to an erroneous diagnosis of cow’s milk allergy in the absence of another explanation.

**Case presentation:**

Our patient, a 48-year-old male with eczema, exercise-induced asthma and rhinoconjunctivitis, presented with four episodes of systemic reactions characterized by mucosal swelling and asthma symptoms following ice-cream exposure. It was later recognized that he had been taking a lactase enzyme supplement just prior to all of his reactions. Epicutaneous testing was strongly positive to a saline slurry of the lactase supplement he had been using. The patient has been avoiding supplemental lactase since with no subsequent reactions.

**Discussion:**

Our patient was diagnosed with an allergy to supplemental lactase enzyme on the basis of convincing Immunoglobulin E (IgE) mediated symptoms and positive skin testing. He continued to eat cow’s milk products, ruling out an IgE-mediated allergy to cow’s milk. In the literature, there is one prior case of anaphylaxis documented. Another case of localized oropharyngeal symptoms described in the literature was thought to be a form of oral allergy syndrome as the patient had positive epicutaneous testing to *Aspergillus oryzae-derived* lactase as well as *Aspergillus* species. Occupational sensitization, rhinitis/asthma, and protein contact dermatitis have also been detailed in the literature. Although rare, this case highlights the importance of a thorough history of over-the-counter supplements when assessing a patient with features of anaphylaxis.

## Background

Lactose intolerance is a common condition characterized by abdominal pain, nausea, bloating, and diarrhea after ingestion of lactase-containing products such as cow’s milk. In most patients, this is due to acquired lactase deficiency caused by a reduction of lactase enzyme production as individuals age [1]. Management may involve avoidance or ingestion of commercially prepared lactase enzyme supplements (bacterial or yeast beta-galactosidases). Allergy to these over-the-counter supplements have been reported in the literature but are rare [2, 3]. Since this supplement is usually taken immediately prior to cow’s milk consumption reactions may be erroneously attributed to cow’s milk allergy. There is only one case of anaphylaxis previously reported in the literature [2]. We present a patient with four episodes of allergic/anaphylactic reactions immediately following the ingestion of a lactase enzyme supplement.

## Case presentation

A 48-year-old Asian male with a history of eczema, exercise-induced asthma, and rhinoconjunctivitis was referred due to concern for new onset cow’s milk allergy. He works as a physician in a hospital/office-based setting and has no known history of Immunoglobulin E (IgE) mediated food allergy (including cow’s milk and tree nut/peanut). His eczema is mild, involving the hands which typically flares in the winter with excess handwashing and a dry/cold environment.

He presented with four episodes of allergic/anaphylactic reactions within 20 min after eating ice cream. The first two reactions were after eating ice cream cake which brought on immediate respiratory symptoms including shortness of breath, chest tightness and wheeze. His third and fourth reactions were similar and occurred after eating nut-free ice cream with immediate onset oral pruritus, oropharyngeal swelling and asthmatic symptoms (shortness of breath, chest tightness and wheeze). His respiratory symptoms were responsive to salbutamol and symptoms resolved over the course of 1–2 h.

On questioning, during the initial consultation, the patient realized he had taken a lactase enzyme supplement prior to each reaction. Non-medical ingredients in the supplement included: included dextrose, microcrystalline cellulose, lactose (50 mg), calcium stearate, and natural mint flavour. He had mild gastrointestinal upset in the past with ice cream so took the supplement as a diagnostic trial. The patient had not consumed lactase enzyme supplements prior to his first reaction however he does handle the lactase enzyme supplements for his son who is lactose intolerant. The patient continued to consume cow’s milk products including ice cream with no issue. He also continued to tolerate tree nuts and peanut which can commonly cross-contaminate ice cream. He consumes peppermint/mint in various forms without issue.

The patient was tested with a saline slurry made from a lactase supplement used prior to his reactions, revealing a positive wheal and flare reaction (6 mm in diameter). Histamine and saline controls were appropriate. Based on a compatible clinical history and skin testing we diagnosed the patient with a supplemental lactase allergy.

We recommended avoidance of lactase supplements, which the patient was agreeable to since lactose intolerance wasn’t a significant issue for him. He has been avoiding lactase supplements since with no further allergic reactions.

## Discussion

Our patient was diagnosed with an allergy to supplemental lactase enzyme following four episodes of systemic/anaphylactic reactions shortly after ingestion. He continued to eat other cow’s milk products, ruling out an IgE-mediated allergy to cow’s milk. Additionally, he had no history of sensitivity to the non-medical ingredients in the supplement. A positive skin test, although not standardized, to the lactase saline slurry was diagnostic in combination with his convincing history. The patient’s latter two reactions do meet criteria for anaphylaxis as per the World Allergy Association updated diagnostic criteria (involvement of mucosal tissue and respiratory compromise) [4].

There are very few cases of IgE-mediated allergy to supplemental lactase in the literature [2, 3, 5]. The only other case of anaphylaxis was reported by Voisin in 2016. The patient developed “bilateral orbital swelling, shortness of breath, and throat constriction after oral ingestion of a supplemental lactase enzyme tablet”. Handling of the lactase enzyme supplement for this patient’s children was thought to be the means of sensitization (as she reacted on her first oral ingestion). In this case the patient also developed contact urticaria with repeated dermal exposure prior to her first systemic reaction. Interestingly the authors report that the patient developed systemic symptoms following skin prick testing requiring treatment with antihistamines and salbutamol.

Similarly, our patient had not orally ingested a lactase supplement prior to his first systemic reaction. We hypothesize that sensitization in our patient’s case occurred via handling of the supplement in the setting of an impaired skin barrier (eczema) or by inadvertent lactase powder inhalation/ingestion when administering the product to his son. This hypothesis is supported by cases of occupational sensitization to lactase enzyme supplements in pharmaceutical workers via direct handling of these products [6]. A single case described a pharmaceutical worker who suffered from contact urticaria, protein contact dermatitis and rhinoconjunctivitis. Her symptoms were temporally related to workplace exposure to lactase supplement dust and she was found to have positive serum lactase-specific IgE testing [5]. Another study found that atopy was a risk factor for positive epicutaneous testing to lactase supplements and that workers who used respirators and “air supplying whole body suits” avoided the development of occupational asthma/rhintitis [6].

Microbial enzymes, including beta-galactosidase (lactase), are high molecular weight sensitizers that are known to cause occupational asthma, rhinoconjunctivitis, and contact dermatitis via respiratory/dermal exposure [7]. Lactase enzymes are produced by the filamentous fungi *Aspergillus oryzae* and *A niger*, a process which is utilized in the pharmaceutical industry for the production of lactase enzyme supplements. An earlier report of confined oropharyngeal symptoms following ingestion of supplemental lactase was proposed to be a form of oral allergy syndrome as the patient had positive epicutaneous testing to *Aspergillus oryzae-derived* lactase as well as *Aspergillus* species [3]. We cannot exclude cross-reactivity between *Aspergillus* and supplemental lactase in our patent as they did not undergo epicutaneous testing to *Aspergillus* species. However, he had repeated systemic reactions characterized by asthmatic symptoms which is an uncommon manifestation of oral allergy syndrome and to our knowledge has not been reported in cases of sensitivity to microbial enzymes [8]. Additionally, if the patient was found to be sensitized to *Aspergillus* this would not change our management. Strict avoidance was advised due to the severity of his reactions which intensified with repeated exposures.

Although rare, this case highlights the importance of a thorough history of over-the-counter supplements when assessing a patient with features of anaphylaxis.

## Data Availability

Data sharing is not applicable to this article as no datasets were generated or analyzed during the current study.
